# Advancing spinal cord injury care through non-invasive autonomic dysreflexia detection with AI

**DOI:** 10.1038/s41598-024-53718-5

**Published:** 2024-02-10

**Authors:** Sidharth Pancholi, Thomas H. Everett, Bradley S. Duerstock

**Affiliations:** 1 Weldon School of Biomedical Engineering, Purdue University, West Lafayette, IN USA; 2 Krannert Cardiovascular Research Center, Division of Cardiovascular Medicine, IU School of Medicine, Indianapolis, USA; 3 School of Industrial Engineering, Purdue University, West Lafayette, USA

**Keywords:** Spinal cord injury, Skin nerve activity, Colorectal distension, Machine learning, Autonomic dysreflexia, Predictive markers, Bone

## Abstract

This paper presents an AI-powered solution for detecting and monitoring Autonomic Dysreflexia (AD) in individuals with spinal cord injuries. Current AD detection methods are limited, lacking non-invasive monitoring systems. We propose a model that combines skin nerve activity (SKNA) signals with a deep neural network (DNN) architecture to overcome this limitation. The DNN is trained on a meticulously curated dataset obtained through controlled colorectal distension, inducing AD events in rats with spinal cord surgery above the T6 level. The proposed system achieves an impressive average classification accuracy of 93.9% ± 2.5%, ensuring accurate AD identification with high precision (95.2% ± 2.1%). It demonstrates a balanced performance with an average F1 score of 94.4% ± 1.8%, indicating a harmonious balance between precision and recall. Additionally, the system exhibits a low average false-negative rate of 4.8% ± 1.6%, minimizing the misclassification of non-AD cases. The robustness and generalizability of the system are validated on unseen data, maintaining high accuracy, F1 score, and a low false-negative rate. This AI-powered solution represents a significant advancement in non-invasive, real-time AD monitoring, with the potential to improve patient outcomes and enhance AD management in individuals with spinal cord injuries. This research contributes a promising solution to the critical healthcare challenge of AD detection and monitoring.

## Introduction

Spinal cord injury (SCI) is a complex medical condition that often leads to various secondary complications, including autonomic dysreflexia (AD), orthostatic hypotension, thermal dysregulation, and bowel and bladder dysfunction^1^. Among these complications, AD is particularly prevalent, affecting approximately 70% of individuals with SCI above the T6 level^2^. AD is characterized by hyperactivity of the sympathetic nervous system in response to a noxious stimulus below the level of injury^3^. Common triggers for AD include urinary bladder overdistension, urinary tract infections, fecal impaction, pressure sores, fractures, menstruation, and various other stimuli^4^. The symptoms of AD vary but often include severe headaches, sweating above the level of injury, flushing or blotching of the skin, nasal congestion, and feelings of anxiety or restlessness. If left untreated, AD can lead to severe complications and even death^5^.

Despite its high incidence, current methods for detecting and monitoring AD are limited, posing significant challenges for both patients and healthcare providers. The gold standard for AD detection is the measurement of a significant increase in systolic blood pressure, typically defined as an increase of 15 mmHg or more^6^. Cuff-based blood pressure monitoring, a common non-invasive technique, presents practical challenges in continuous AD management. Its intermittent nature requires manual operation, leading to gaps in real-time data collection and making immediate AD detection difficult. Patients are burdened with the constant need for monitoring, disrupting their daily activities and diminishing their quality of life. To address these limitations, the development of non-invasive, continuous monitoring systems is essential. These advancements will not only enhance AD management but also provide patients with a more seamless and unobtrusive monitoring experience. Previous research has utilized animal experimentation, particularly using rats, to understand the behavioral manifestations of AD in response to different scenarios and stimuli^7–9^. These studies have provided valuable insights into the disease’s progression, shedding light on underlying mechanisms and facilitating the development of strategies for managing AD in humans.

Artificial Intelligence (AI) has emerged as a powerful tool in healthcare, offering accurate and real-time insights to healthcare professionals^10,11^. The advent of wearable sensing technologies has generated large volumes of data that can be leveraged by AI-powered solutions to detect diseases early and develop personalized treatment plans based on individual data. AI-based decision support systems can assist healthcare professionals in making informed decisions by analyzing vast amounts of data from various sources, such as electronic health records and physiological data^12^. Wearable sensors, including electrocardiogram (ECG), photoplethysmography (PPG), electroencephalogram (EEG), and electromyogram (EMG), have demonstrated accurate detection of abnormalities in various physiological signals^13–15^. These sensors can be integrated into wearable devices, such as smartwatches, wristbands, and patches, enabling continuous monitoring of an individual’s physiological signals.

Accurate detection of in individuals with SCI relies on extracting relevant features from the input signals to enhance the predictive ability of machine learning models^16^. Feature extraction involves identifying important patterns and characteristics within the signals, enabling the algorithm to make accurate predictions. Time domain features are particularly advantageous as they can be directly calculated from the signals without requiring complex transformations^17,18^. Various machine learning algorithms, including Decision Trees, Support Vector Machines (SVM), Linear Discriminant Analysis (LDA), K-Nearest Neighbors (KNN), and Random Forest (RF), have traditionally been employed to train and predict physiological signal episodes related to AD. However, Deep Neural Networks (DNN) have gained popularity due to their ability to outperform other algorithms and adapt to changing parameters^17,19^. Neural networks have demonstrated remarkable effectiveness in disease diagnosis, especially in tasks such as disease classification and prediction. By leveraging pathological or physiological data, neural networks allocate greater weight to important features, enabling accurate categorization of diseases and insightful predictions^20^. Through the training process, neural networks autonomously learn to assign higher significance to relevant aspects of the data, enhancing their understanding of the underlying patterns. Their ability to intelligently analyze diverse facets of the input enables neural networks to excel in identifying crucial patterns and providing precise classifications or predictions. As a result, they significantly contribute to effective disease diagnosis and drive advancements in healthcare outcomes.

Recent research has focused on developing a self-monitoring physiological telemetry system for tetraplegics, utilizing smart watch sensors to monitor physiological indicators of AD in real-time throughout the day^21^. In rat models with SCI, specific time domain features of SKNA have proven highly effective in detecting AD. This achievement, documented in studies^22,23^, demonstrates remarkable accuracy levels. Monitoring the sympathetic nervous system becomes crucial in the context of AD detection, a condition where there is an exaggerated response of the autonomic nervous system to a noxious stimulus, often occurring in individuals with spinal cord injuries^24^. The sympathetic branch, functioning as an accelerator, plays a significant role in this heightened autonomic activity. Examining sympathetic nerve activity (SNA) becomes instrumental for diagnosing and stratifying the risks associated with AD, captivating the attention of both patients and healthcare providers^25^. In contrast to invasive SNA measurement methods, SKNA emerges as a non-invasive means to assess nerve activity, particularly from the stellate ganglion. The stellate ganglion, located in the neck region, assumes a central role in regulating sympathetic nerve signals linked to skin blood flow^26^. Understanding its influence becomes paramount in deciphering the intricate relationship between sympathetic nerve activity, skin responses, and the manifestation of AD. This knowledge stands as a cornerstone in unraveling the physiological implications of autonomic nervous system dynamics in the context of this condition.

These advancements demonstrate the potential of AI and neural networks in disease diagnosis, classification, and prediction tasks, and their ability to significantly contribute to improved healthcare outcomes. One significant challenge that arises from the occurrence of unexpected spikes in nerve activity during the baseline is the difficulty it poses in accurately classifying AD events versus non-AD events. These sporadic spikes can complicate the identification and differentiation between normal and pathological nerve activity patterns, making it more challenging to distinguish AD-related signals from other non-AD-related signals.

By addressing the limitations of current AD detection methods and leveraging the power of AI and wearable sensing technologies, this study aims to develop an innovative approach for the early and continuous detection of AD in individuals with SCI. This research will contribute to enhancing the management and treatment of AD, ultimately improving the quality of life for individuals living with SCI. The paper proposes a DNN architecture for accurately detecting AD events using only the SKNA signal. The DNN model was trained on labeled data sets and outperformed previous methods that utilized multiple physiological features, demonstrating that SKNA alone is sufficient for detecting AD-related changes in the autonomic nervous system. The model’s performance was evaluated on 30% of unseen data, confirming its robustness and ability to generalize well. These findings suggest that the proposed DNN model has the potential for clinical applications in AD detection.

**Figure 1:**
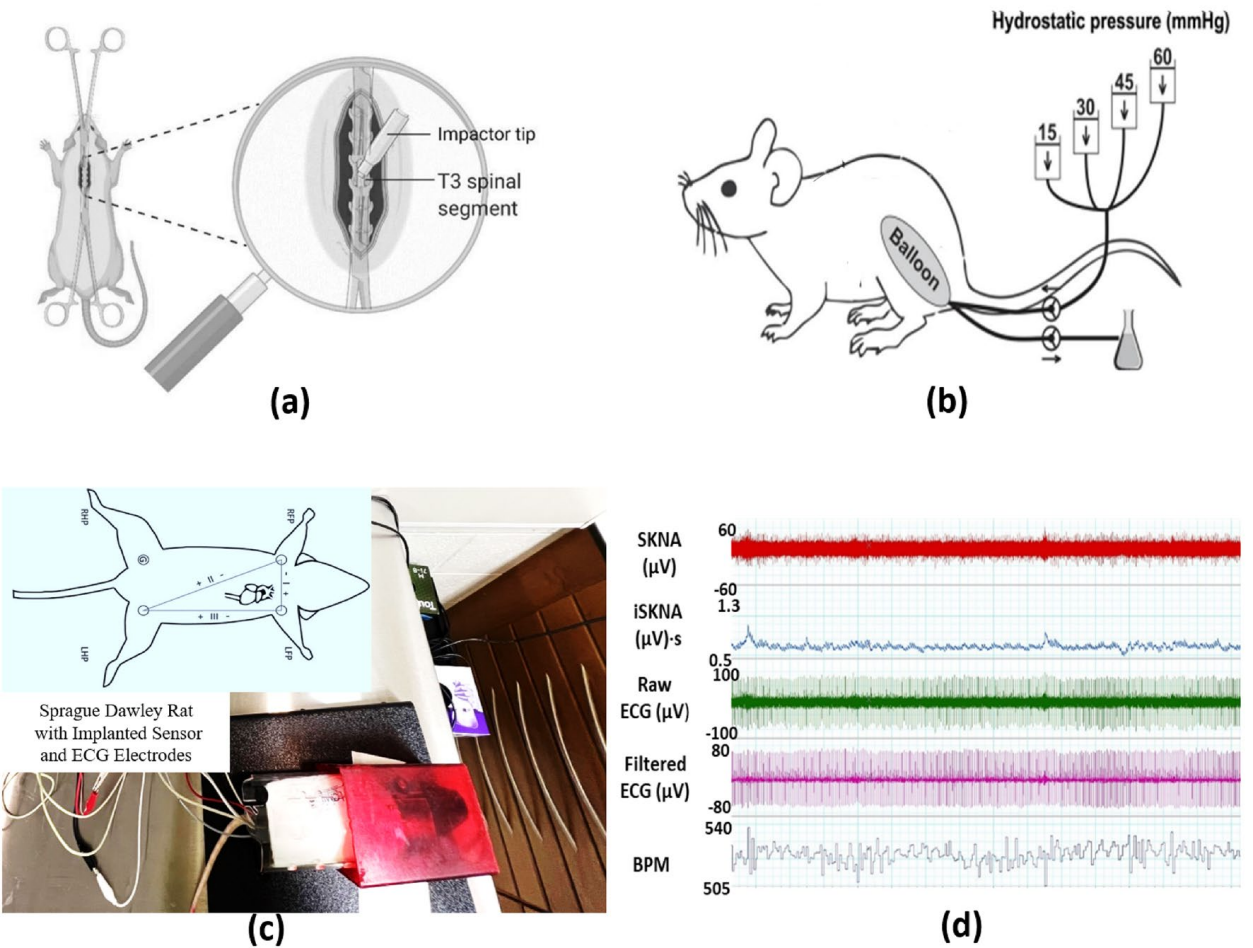
(**a**) This figure illustrates the experimental SCI procedure conducted above the T6 level of the spinal cord, providing a detailed view of the targeted thoracic vertebra 3 (T3). (**b**) AD was induced through CRD, employing an infla Foley catheter inserted into the rectum, mimicking physiological stress. (**c**) Conscious rats were carefully restrained within the experimental setup, ensuring secure affixing of electrodes for non-invasive data recording, including Skin Nerve Activity (SKNA), Integrated Skin Nerve Activity (iSKNA) which refers to Integrated Skin Nerve Activity, a measurement that combines various aspects of skin nerve responses over a specific time frame, Electrocardiogram (ECG), Blood Pressure (BP), and Heart Rate (HR). (**d**) The acquired data encompassed physiological parameters crucial for studying autonomic responses and understanding sympathetic nervous system activation in response to external stimuli.

## Methods

### Data acquisition

Fifteen male Sprague Dawley rats^27^, were purchased from Envigo (Indianapolis, IN) and implanted with a single pressure and biopotential implant (HD-S11 Implant, DSI International, USA) to record blood pressure and ECG in real-time with a sampling rate of 1 kHz. Male rats were selected exclusively for the study due to the high incidence of SCI in men, who account for around 80% of all cases and tend to sustain injuries at an earlier age. Additionally, non-invasive equipment was used to record SKNA and ECG at a sampling rate of 10kHz^22^. The animals were housed individually in a Plexiglas cage with straw bedding and were provided with ad libitum access to food and water. After a four-week acclimation period to the experimental setup and sensors, including electrodes, a restraining jacket, and a plastic holder with air holes (HLD-RL model, Kent Scientific, USA), each rat underwent a dorsal laminectomy followed by a spinal cord crush with specially designed forceps. This resulted in the loss of motor function in both hind legs of the rats, and a recovery period of five days was allowed before experimentation. Figure 1 showcases the experimental configuration and the diverse signals obtained from a rat, providing an insightful demonstration of the study.

For each experiment, ECG electrodes were placed on the rats in the Lead I configuration, and the rats were placed in a restraining jacket that was subsequently placed in a restraining tube^23,28^. In our experimental approach focused on understanding the activation of the sympathetic nervous system, we utilized a technique called Colorectal Distension (CRD). This method involves inducing controlled pressure within the rectum by inflating a balloon, simulating distension and discomfort. During our study, CRD played a pivotal role as we performed this procedure for 1-min intervals, three times over the course of the entire 50-min experiment. The purpose behind employing CRD was to deliberately trigger AD, characterized by heightened activity in the sympathetic nervous system. To induce AD, a balloon catheter was inserted into the rectum, and we recorded a 20-min baseline data period. Following this, at 10-min intervals, the catheter was inflated for 1 min, with this cycle repeated three times. This carefully controlled experimental setup allowed us to gain valuable insights into the intricate autonomic responses associated with AD. The protocol used for this study was approved by the Purdue University Animal Care and Use Committee. Overall, this experimental setup allowed for the simultaneous recording of multiple physiological parameters, including blood pressure, ECG, and skin nerve activity, providing a comprehensive data set for the analysis of AD in rats. This project has received approval from the Purdue Animal Care and Use Committee (PACUC) with the assigned approval number being 1810001814. Our study was also carried out in compliance with the ARRIVE guidelines (http://www.nc3rs.org.uk/page.asp?id=1357). All methods were conducted in accordance with relevant guidelines and regulations.

### AD and Non-AD segmentation from SKNA recording

An implanted sensor simultaneously recorded arterial BP as the gold standard for determining AD events before, during, and after CRD procedures. AD events were identified based on the presence of systolic blood pressure exceeding 15 mmHg accompanied by bradycardia^29^.

**Figure 2:**
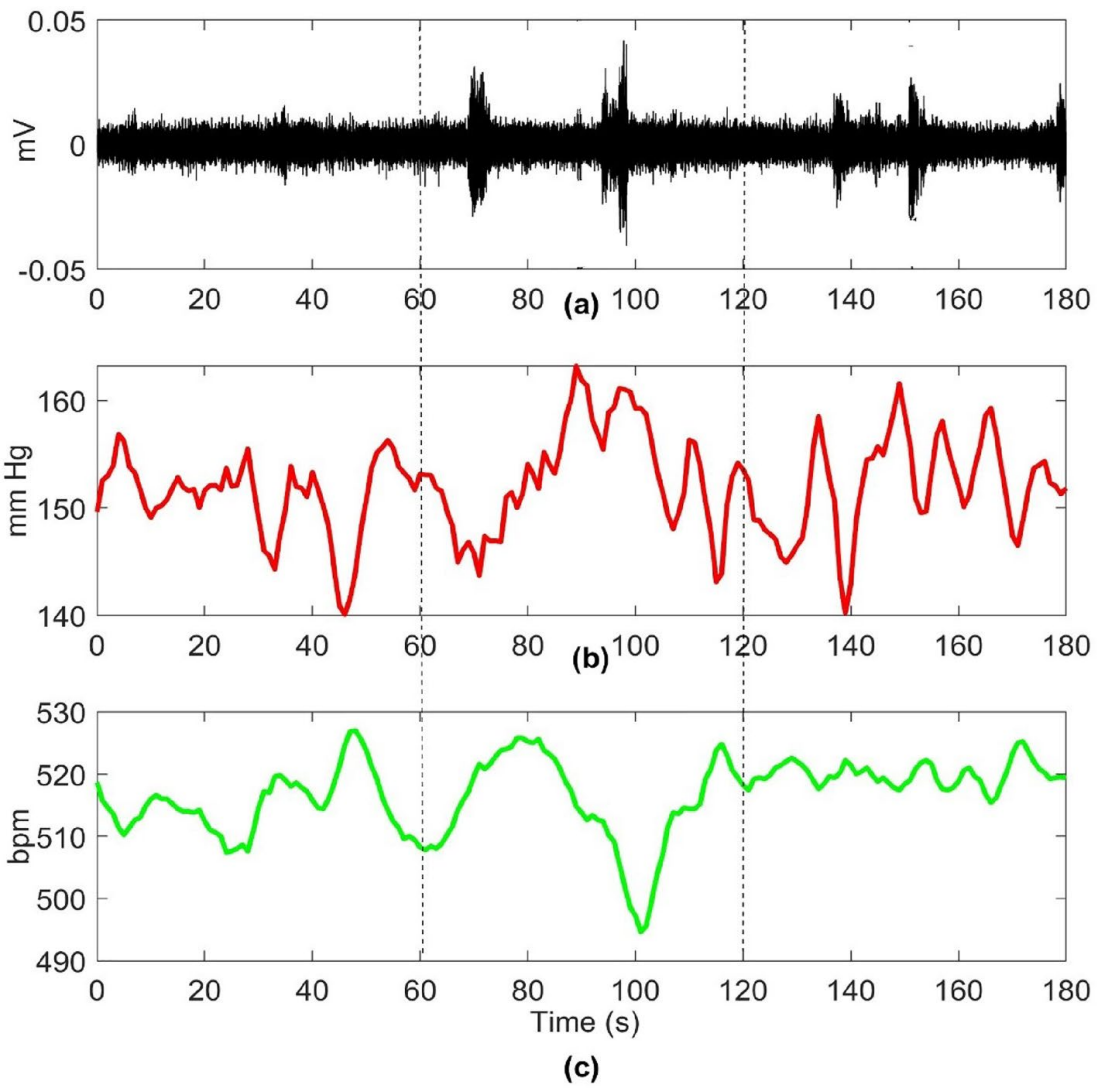
(**a**) A SKNA signal during baseline and during CRD (inside dotted lines) with the induction of an AD event, (**b**) Blood pressure measurement (**c**) heart rate (HR) with an immediate decrease observed after the rise in blood pressure.

To develop a robust machine learning model, the SKNA signal was meticulously partitioned into segments corresponding to AD and non-AD events. Each AD event, lasting precisely 1 min, was carefully delineated. It’s essential to note that these measurements originated from the exact same location on the same animal throughout the experiments. AD signal segments were specifically derived when AD was successfully induced, indicated by a blood pressure increase beyond 15 mm Hg, triggered by CRD. Conversely, non-AD signal segments were selected from trials where CRD was not performed. These non-AD events were randomly chosen from the baseline recording, encompassing potential SKNA spikes due to factors like hypertension or other variables in the rat model. These selected non-AD events from the baseline were utilized for training the machine learning model, ensuring its capability to handle real-world challenges such as noise and motion artifacts. Figure 2 illustrates an AD event that occurred during the performance of CRD. The baseline recording showed a single spike, while several SKNA bursts were observed during the CRD.

**Table 1:** Feature descriptions and formulas.

Feature	Description	Formula
Variance	Measures the spread of the signal around its mean value, providing information about the signal’s variability	Variance = $\frac{1}{N} \sum (x - \mu )^2$
Kurtosis	Measures the peakiness of the distribution of the signal’s amplitude, providing information about the signal’s distribution	Kurtosis = $\frac{1}{N} \sum \left( \frac{x - \mu }{\sigma }\right) ^4 - 3$
RMS	Calculates the root mean square of the signal, representing the signal’s power and energy	RMS = $\sqrt{\frac{1}{N} \sum (x^2)}$
WL	Provides information about the signal’s shape and frequency content by measuring the cumulative length of the signal’s waveform	WL = $\sum |x(i) - x(i-1)|$
ZC	Counts the number of times the signal crosses the zero axis, providing information about the signal’s frequency	ZC = $\frac{1}{2} \sum |sign(x(i)) - sign(x(i-1))|$
SSC	Counts the number of times the slope of the signal changes sign, providing information about the signal’s rate of change	SSC = $\sum (\text {sign}(\text {diff}(x)) \ne 0)$
WAMP	Calculates the weighted sum of the absolute differences between adjacent samples that exceed a threshold value, providing information about the signal’s amplitude changes over time	WAMP = $\sum (|x(i) - x(i-1)| > T)$
Crest Factor	Measures the peak-to-average ratio of the signal, providing information about the signal’s dynamic range	Crest Factor = $\frac{\text {Peak Value}}{\text {RMS Value}}$

*N* denotes the number of samples, *x* represents the sample value, $\mu$ is the mean value of the signal, $\sigma$ is the standard deviation of the signal, *x*(*i*) denotes the ith sample value of the signal, *T* represents the threshold value, and sign() and diff() are the sign and difference functions, respectively.

### Signal pre-processing and feature extraction

The signal processing approach involved bandpass filtering the time series data between 500 and 1000 Hz using an 8th-order Butterworth filter to remove any unwanted noise or artifacts that could negatively impact the feature extraction process. The resulting filtered signal was then normalized to the range − 1 to 1, which is a common and effective pre-processing step in the signal analysis^30^.

In a previous study, five potential SKNA signal biomarkers for AD detection were identified, including medianNN, average iSKNA, number of bursts, RMSSD, and pNN5^16^. However, we discovered that the RMSSD and pNN5 features were delayed in detecting AD, and identifying bursts in the iSKNA signal was difficult due to varying thresholds based on amplitude^31^. To overcome these limitations, we decided to focus exclusively on the SKNA signal and selected features that were not dependent on specific thresholds. The selected features for the proposed model, such as Variance, Kurtosis, RMS, WL, ZC, SSC, WA, and CF, capture different aspects of the signal’s characteristics^32,33^. They provide information about the signal’s variability, distribution, power, waveform, frequency content, rate of change, amplitude changes, and dynamic range as defined in Table 1. The combination of these features offers a diverse set of information about the skin nerve activity signals^34^. By capturing different aspects of the signals, they provide a comprehensive representation that helps the DNN model learn relevant patterns and discriminative features. We used an overlapping window technique with a window length of 5 s and a 0.5-s overlap to derive the features from the SKNA signals. This ensured the accuracy and robustness of the extracted features that truly represented the desired behavior in the signals as shown in the Fig/ 3^35^.

**Figure 3:**
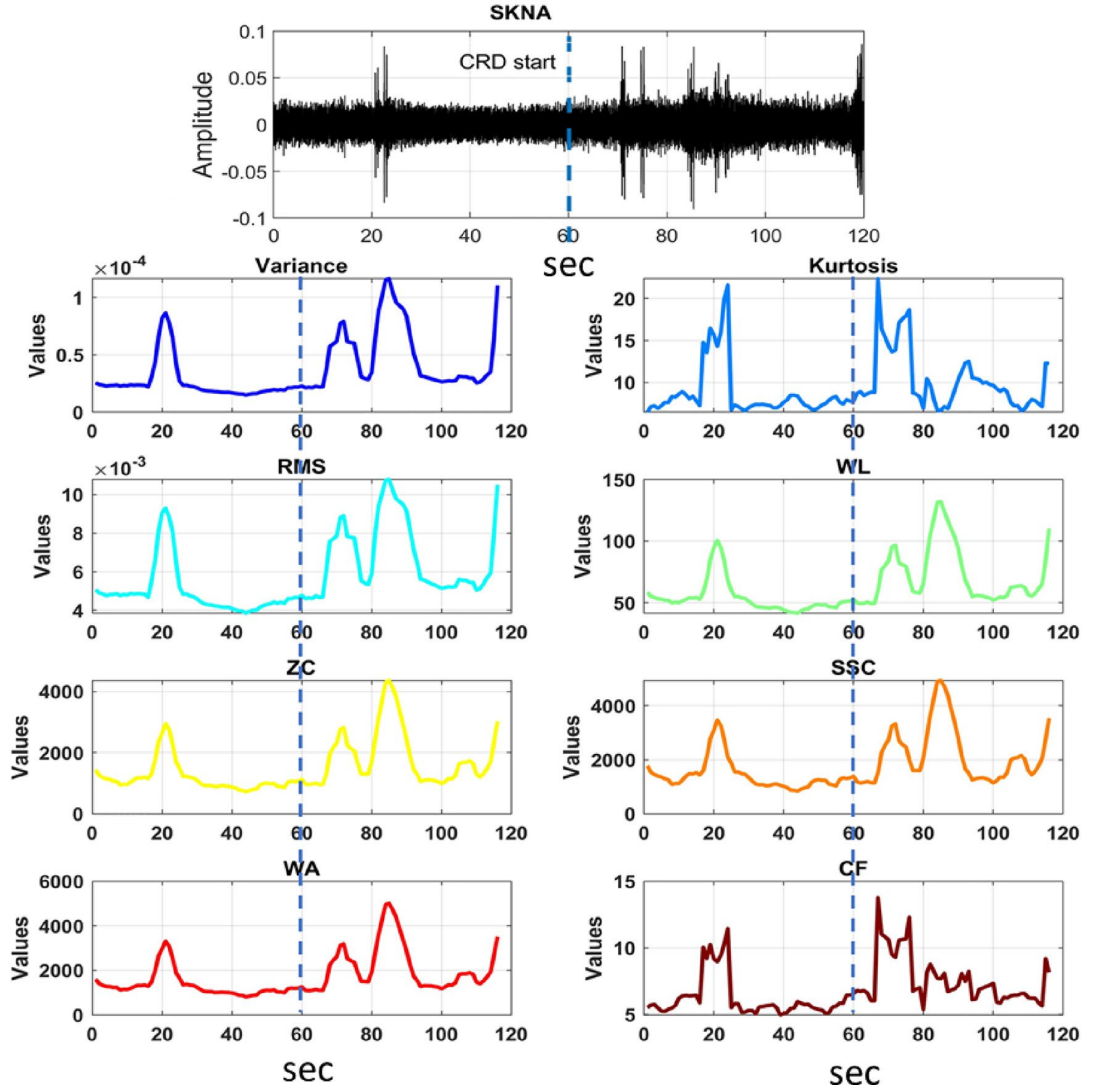
Comparison of baseline signals and an AD event after CRD (see vertical dotted line), along with all the features that were extracted from the data during this course. These visual representations of how the different features vary over time and in response to the AD event.

**Figure 4:**
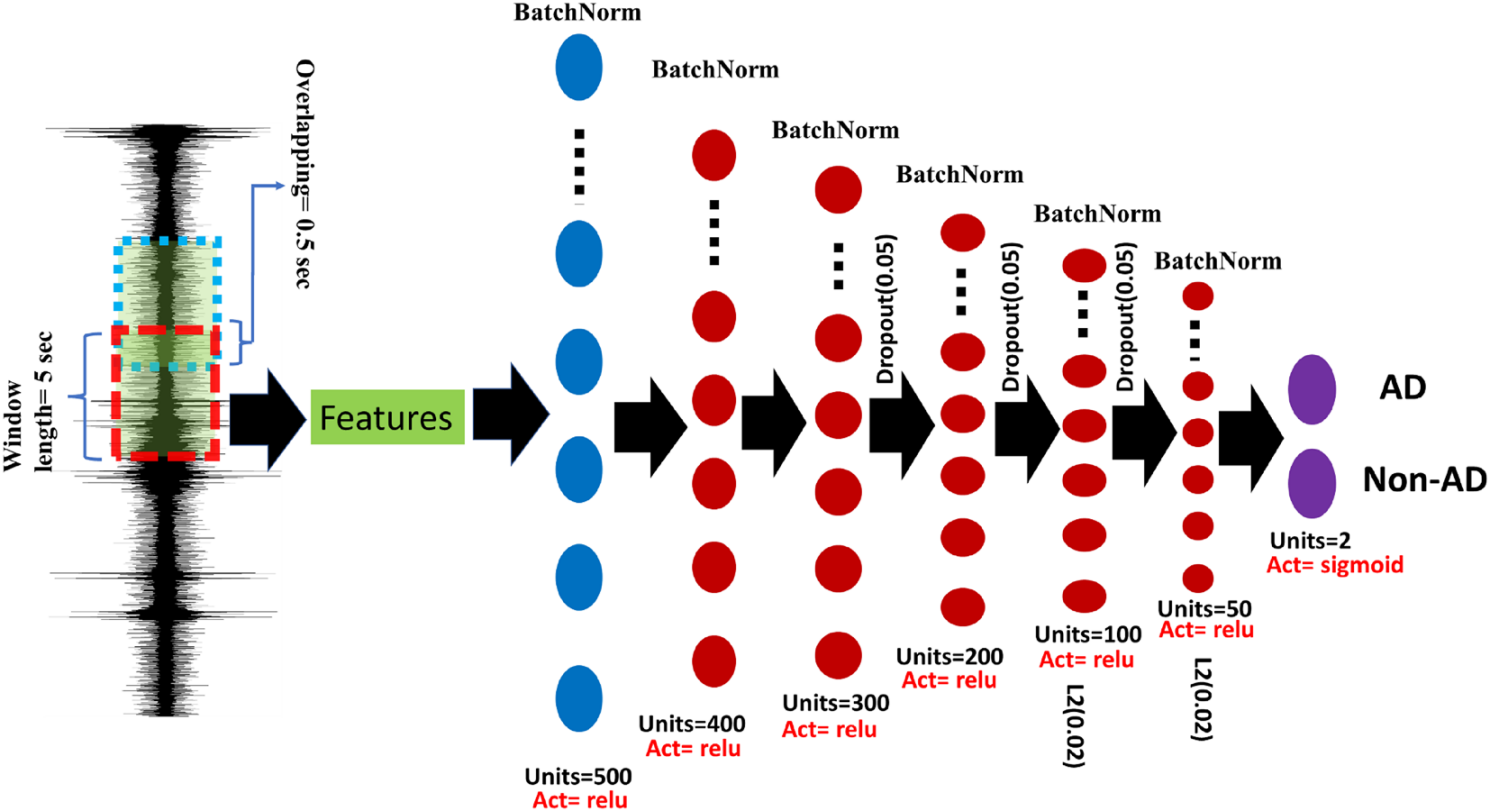
This illustrates the neural network architecture for detecting AD and Non-AD events. The various layers and connections between them enabled the model to classify the input data.

### Classification algorithm and analysis matrices

DNNs have been found effective in diagnosing diseases by classifying biological signals^36,37^. The proposed DNN architecture includes six dense layers using varied numbers of neurons and regularization techniques as shown in Fig. 4. The first dense layer has 500 neurons and accepts the training features’ input shape. Batch normalization was applied after each dense layer to normalize the input for the following layer. The second dense layer had 400 neurons, followed by the third dense layer with 300 neurons. The fourth and fifth dense layers have 200 and 100 neurons, respectively. The final dense layer had 50 neurons, applying L2 regularization with a parameter of 0.02 and using the sigmoid activation function for binary classification. To avoid overfitting, dropout regularization with a 0.05 rate was applied after the third and fourth dense layers. The model was compiled with binary cross-entropy loss and stochastic gradient descent optimizer. The model was trained with 500 epochs using a batch size of 32 for training data, with validation data provided during training. The model’s weights were saved with the best validation accuracy using a checkpoint callback. By utilizing DNN techniques, this approach provided an effective tool for automated AD diagnosis from SKNA signals.

To evaluate the performance of the proposed model, commonly used metrics such as True Positives (TP), False Positives (FP), True Negatives (TN), False Negatives (FN), Precision, Recall, F1-score, Sensitivity, and Specificity were used [ref needed]. TP represents correctly classified positive samples, FP represents negative samples incorrectly classified as positive, TN represents correctly classified negative samples, and FN represents positive samples incorrectly classified as negative. Precision is the fraction of true positives out of all samples classified as positive, while Recall is the fraction of true positives out of all actual positive samples. The F1-score is the harmonic mean of precision and recall. Sensitivity represents the true positive rate, or the fraction of actual positive samples that are correctly classified as positive, while Specificity represents the true negative rate or the fraction of actual negative samples that are correctly classified as negative. These metrics will help in assessing the model’s ability to distinguish between AD and non-AD events.

## Results

**Figure 5:**
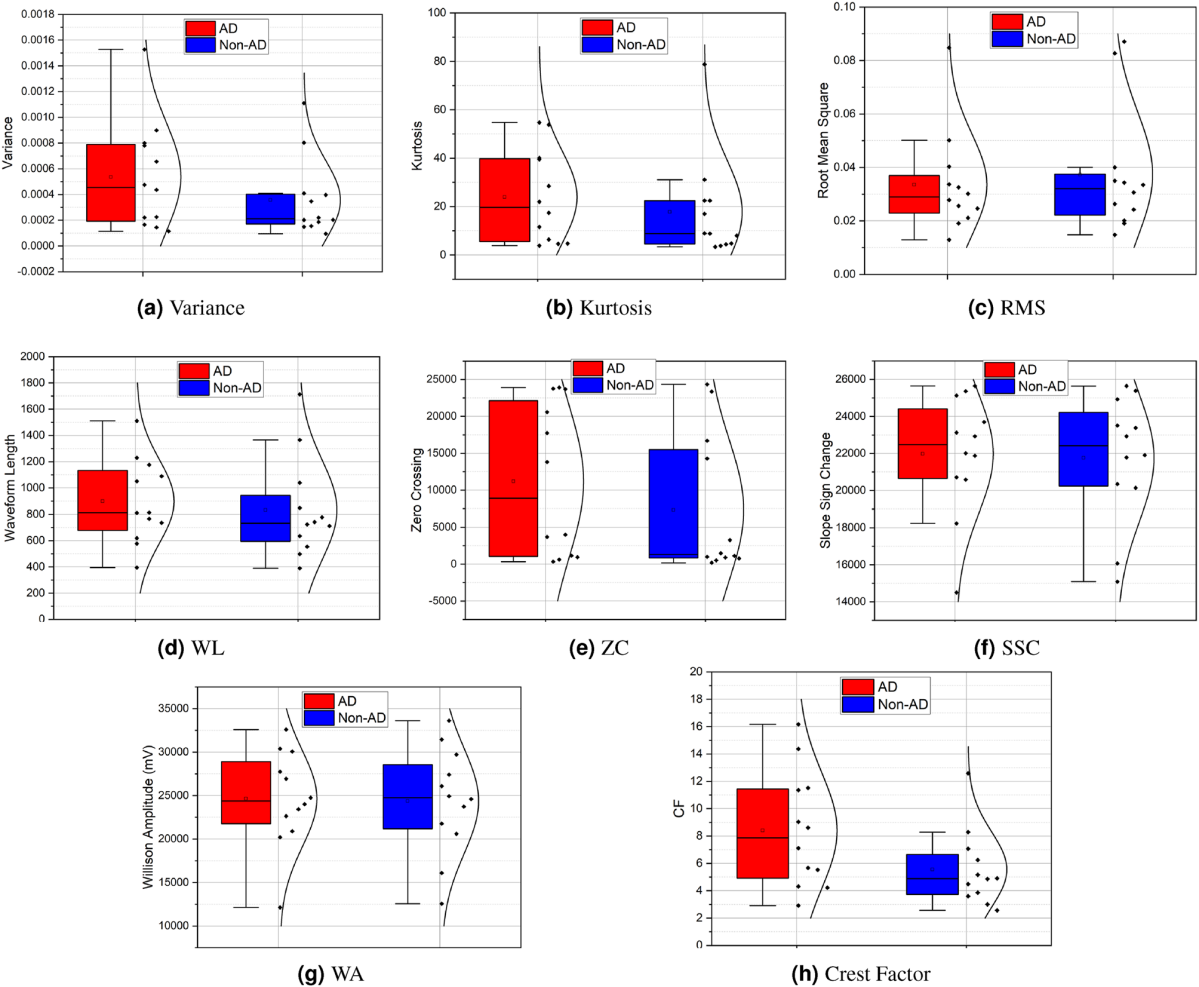
Graphs illustrating box plots of distribution of features for AD and non-AD.

In the result section, we present the findings of our analysis, demonstrating the significance of each extracted feature in distinguishing AD events from non-AD events. Each feature provides unique information, and our statistical metrics reveal distinct patterns between the two groups. Variance, Kurtosis, Root Mean Square (RMS), Waveform Length (WL), Zero Crossings (ZC), Slope Sign Change (SSC), Amplitude-Waveform Area (WA), and Crest Factor (CF) were among the key features analyzed, highlighting their potential impact on the classification of AD events as shown in Fig. 5. These results contribute to a deeper understanding of AD and pave the way for potential diagnostic advancements.

### Feature analysis

We calculated various statistical metrics for each feature, including maximum, mean, minimum, and standard deviation, to understand the differences between the two groups. Our results revealed distinct patterns in the analyzed features between the AD and Non-AD groups. Firstly, in terms of Variance, the AD group exhibited higher values (maximum: 0.001528, mean: 0.000463) compared to the Non-AD group (maximum: 0.0008, mean: 0.000339), indicating increased variability in the skin nerve activity signals among individuals with AD. Similarly, the Kurtosis values were higher in the AD group (maximum: 54.71572, mean: 25.625879) compared to the Non-AD group (maximum: 39.480322, mean: 22.41112), suggesting more pronounced peakiness and heavier tails in the distribution of the nerve activity signals for individuals with AD. Moving on to RMS, the AD group had higher values (maximum: 0.087063, mean: 0.053605) compared to the Non-AD group (maximum: 0.084781, mean: 0.034548), indicating larger overall amplitudes in the skin nerve activity signals among individuals with AD. Similarly, the WL values were higher in the AD group (maximum: 1229.583535, mean: 874.294221) compared to the Non-AD group (maximum: 811.722025, mean: 657.936163), suggesting more complex and intricate waveforms in the nerve activity signals of individuals with AD. Furthermore, the ZC values were higher in the AD group (maximum: 23919.58824, mean: 17788.39685) compared to the Non-AD group (maximum: 17730.47059, mean: 15530.09685), indicating a higher frequency of changes in signal polarity in the nerve activity signals for individuals with AD. Similarly, the SSC values were slightly higher in the AD group (maximum: 25649.76471, mean: 22915.20846) compared to the Non-AD group (maximum: 25129.41177, mean: 22552.04408), suggesting more frequent changes in signal slope in the nerve activity signals for individuals with AD.

The WA values were higher in the AD group (maximum: 32596.2353, mean: 26062.50403) compared to the Non-AD group (maximum: 30388.76471, mean: 24471.86757), indicating larger amplitudes in the nerve activity signals for individuals with AD. Lastly, the CF values were higher in the AD group (maximum: 16.170041, mean: 10.15679) compared to the Non-AD group (maximum: 14.36859, mean: 8.82605), suggesting sharper peaks in the waveform of the skin nerve activity signals for individuals with AD.

Additionally, the Multivariate analysis of variance (MANOVA) results reveal significant differences between the groups in the multivariate space (p< 0.05). The intercepts for Wilks’ lambda, Pillai’s trace, Hotelling-Lawley trace, and Roy’s greatest root were 0.0086, 0.9914, 115.9416, and 115.9416, respectively. These values indicate substantial group separation. The corresponding p-values were all 2.49111e-15, underscoring the statistical significance of these differences. This suggests that the overall multivariate means of the groups are significantly different. The analysis reaffirms that the examined variables collectively discriminate between AD and Non-AD groups, emphasising the robustness of our findings.

**Figure 6:**
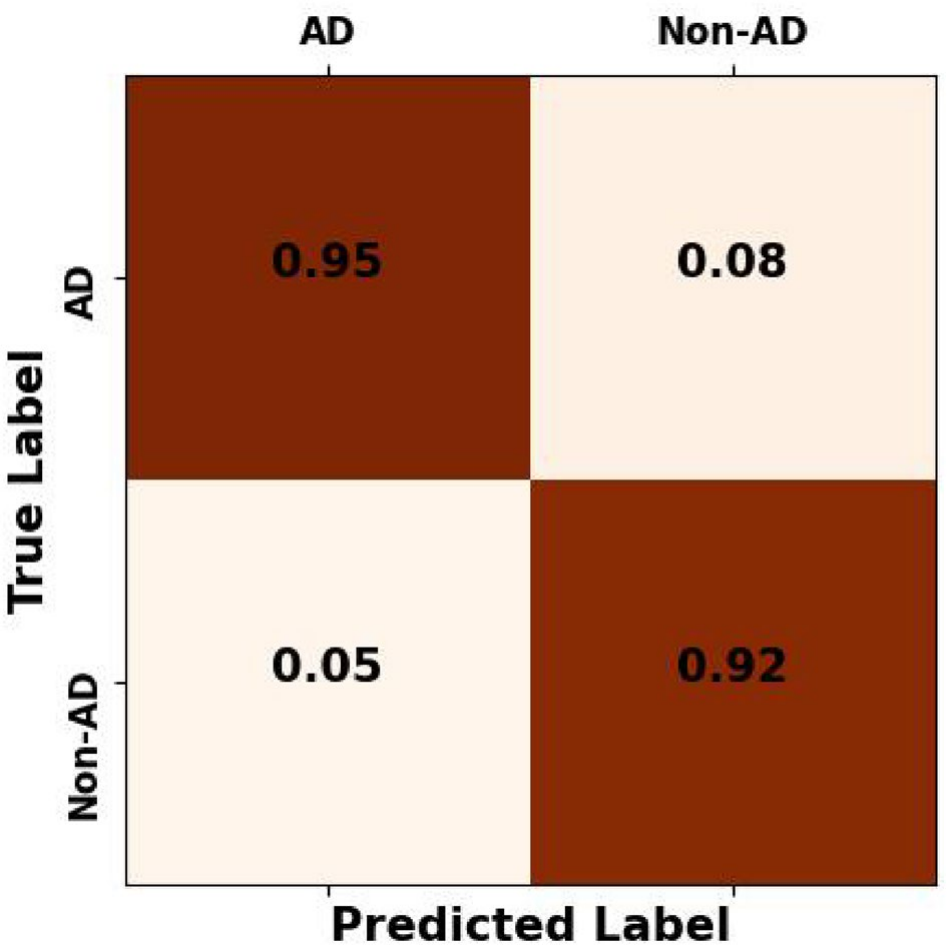
The mean confusion matrix across rats exhibits an overall false-negative rate of 0.05, encapsulating the collective performance of the models in detecting autonomic dysreflexia events.

### Classification performance

**Figure 7:**
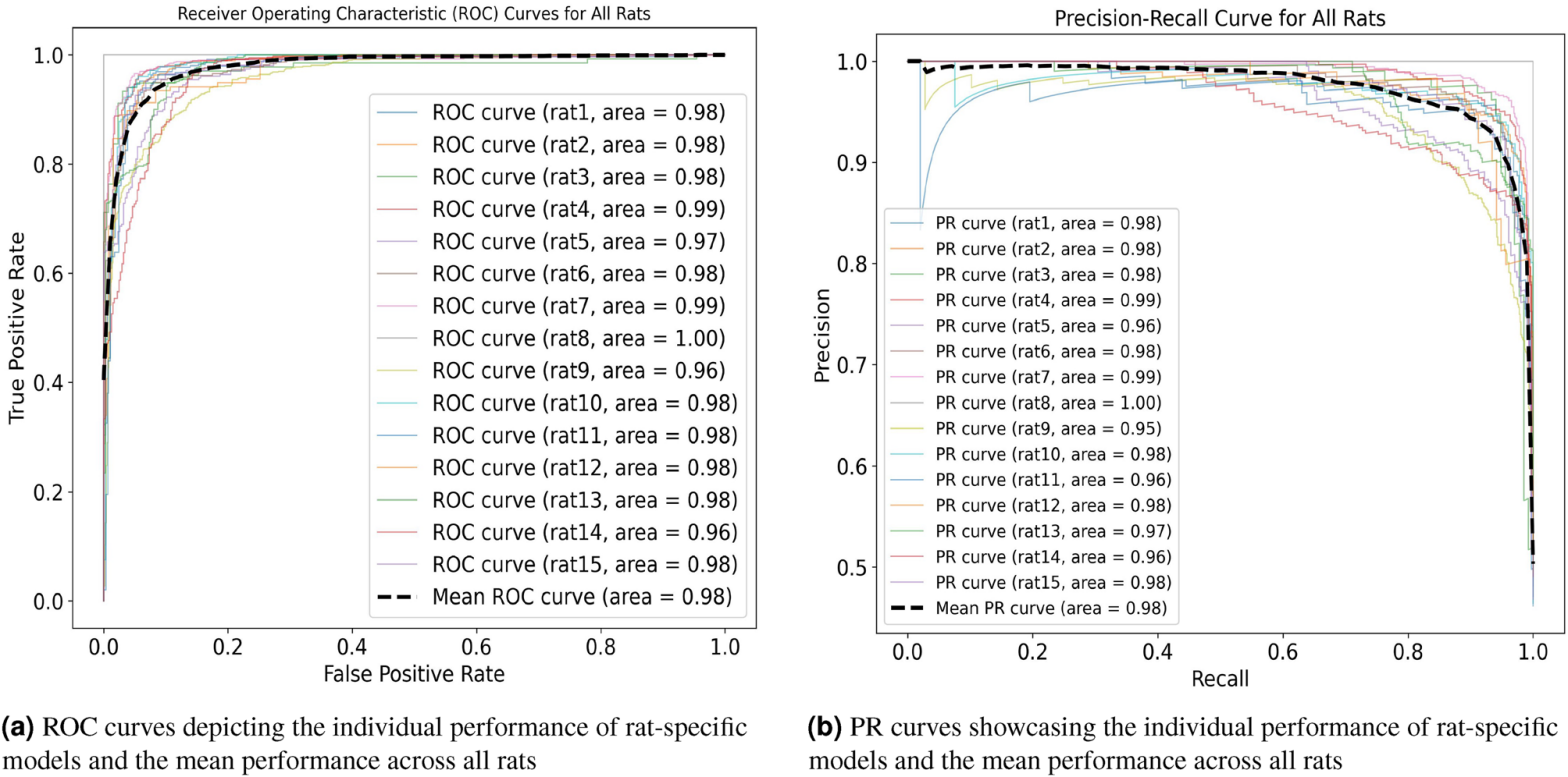
ROC and PR curves to understanding the performance of rat-specific models, providing clarity on their discrimination ability and precision-recall trade-offs.

First and foremost, the model exhibited exceptional accuracy in identifying AD cases, achieving an average accuracy of 93.9% (±m 2.5%). This indicates that the model was highly proficient in differentiating between AD and non-AD cases, with a relatively low error rate. These findings suggest that the DNN model holds significant promise as a robust tool for AD detection in rats induced by CRD. To further evaluate the model’s performance, we employed the F1-score, a metric that balances precision and recall. The average F1-score obtained was 0.944 (±0.018), indicating a commendable balance between accurately identifying true positive AD cases and minimizing false positives and negatives. This high F1-score underscores the model’s ability to balance capturing AD cases and avoiding misclassification. Examining the false negatives, we found an average value of 4.8% (± 1.6%). This implies that the model incorrectly classified a small proportion of non-AD cases as AD. While this rate is relatively low, it does highlight the importance of further improving the model’s sensitivity to ensure the accurate identification of all AD cases. Nevertheless, it is worth noting that the model consistently achieved an impressive average true positive rate of 95.2% (± 2.6%), demonstrating its effectiveness in capturing the most true positive AD cases. Additionally, the model demonstrated high precision and recall, with average values of 95.2% (± 2.1%) for both metrics. This indicates the model’s ability to achieve high precision in correctly identifying AD cases and high recall in capturing most of the AD cases among all positive predictions. These results further validate the model’s efficacy in accurately detecting AD cases induced by CRD in rats. The observed standard deviations in accuracy, F1-score, false negatives, true positive rate, precision, and recall reflect the variability in model performance across different rats. They also underscore the importance of considering individual variations in the interpretation of the results. However, it is noteworthy that the relatively small standard deviations suggest consistent and stable model performance. This stability is indicative of the model’s robustness and reliability, as its accuracy appears to be less influenced by rat-specific factors. The mean confusion matrix for all rat models’ performance is presented in Fig. 6. The Fig. 7a,b showcase Receiver Operating Characteristic (ROC) and Precision-Recall curves, providing insights into the discriminative power and precision-recall trade-offs of rat-specific models. The mean ROC-AUC is reported as 0.98, indicating a high overall discriminative capability. Each ROC curve delineates the model’s ability to distinguish between classes, with the bold line representing the mean performance across all rats. Correspondingly, Precision-Recall curves offer a detailed perspective on individual and aggregate precision and recall rates, with a mean PR-AUC of 0.98. Together, these metrics provide a comprehensive assessment of the models’ efficacy in the predictive framework.

## Discussion

To ensure the reliability and validity of our results, we meticulously reviewed our data and found that three rats had passed away prematurely prior to the completion of the experiment. Consequently, we have appropriately excluded these rats from our analysis to ensure that our findings are robust and accurately reflect the intended population. The DNN model demonstrated high precision and recall values for the majority of rats, indicating a low rate of false positives and a high detection of true positives. The F1 scores further supported the model’s overall efficiency in identifying true positives. Additionally, the sensitivity and specificity values demonstrated a high level of accuracy in detecting both true positives and true negatives. The model showed promising results, and the false negative rate was relatively low for most rats, suggesting that the model could be a solution for AD detection. The lowest accuracy achieved was 92.2%, indicating its ability to correctly identify the majority of AD cases. This result underscores the model’s efficacy in accurately classifying AD cases, even in challenging circumstances. Furthermore, the lowest F1 score achieved was 93.6%, representing a remarkable balance between precision and recall. This score highlights the rat parameter’s ability to achieve a high level of precision in correctly identifying AD cases while effectively capturing a significant proportion of true positive AD cases among all positive predictions. We employed scatter plots to analyze the classification performance of AD and baseline episodes in rats subjected to CRD over a duration of 1 min. However, we encountered a challenge as certain features of AD and baseline episodes exhibited overlapping patterns, which hindered the use of linear classification methods.

We observed that AD episodes displayed distinct characteristics regarding SKNA activity distribution, neuron spiking, and signal amplitude or power. Notably, AD episodes exhibited a pronounced increase in these aspects compared to baseline episodes. This was evident in scatter plots as shown in supplementary figure 1, such as Kurtosis vs. RMS or RMS vs. SSC, where a substantial number of observations clustered in regions associated with heightened neuronal activity. The clustering of non-Ad events suggests that they tend to occur near each other, while AD events are scattered and spread out widely. The SSC analysis indicates that when an AD event occurs, there are more neuronal firings originating from the stellate ganglion. This observation is consistent with the ZC vs RMS plot, where the occurrence of a burst leads to an increased number of ZCs. Examining the shape or kurtosis feature reveals an interesting pattern. During non-AD events, the kurtosis values are distributed closely together. However, when an AD event occurs, the kurtosis values are spread out further and are not tightly distributed. This suggests that the shape and duration of the bursts can vary significantly during AD events. The correlation matrices for both AD and Non-AD features indicate sparse interconnections among variables. In the AD features, only a few pairs, such as ’SSC’ and ’WA’, exhibit a substantial positive correlation (0.97), indicating limited feature interdependence. Similarly, the Non-AD features show minimal correlations, emphasizing the absence of strong relationships between variables. Despite this, removing these correlated features might significantly reduce the feature matrix size, potentially impacting model performance, (presented in the supplementary figure 2).

Our research excels previous work in prompt AD detection using AI. While past studies used a 15-s time window for feature extraction from iSKNA signals^16^, our method employs a more efficient 5-s window. This advancement enhances the speed and accuracy of AD detection, showcasing the evolution of AI in medical research.

## Conclusion and future work

This paper presents an AI-powered solution for the detection and monitoring of AD in individuals with SCI using non-invasive sensors to measure changes in sympathetic activity. The proposed system overcomes the limitations of current AD detection methods and blood pressure monitoring systems by utilizing the skin nerve activity signal and deep neural network architecture with high precision, high recall, and low false-negative rates. The system’s robustness and ability to generalize well were confirmed by evaluating its performance on naïve data. This research represents a significant advancement towards non-invasive, real-time monitoring of AD that may be translatable to humans with SCI. The potential for early detection and timely intervention using this AI-powered solution before AD symptoms dangerously escalate could significantly enhance patient outcomes and improve the management of AD. Further, we aim to broaden our study’s scope to enhance early detection. Our extended research will introduce a grading system quantifying Autonomic Dysreflexia’s severity and early symptoms. Furthermore, human experiments will incorporate additional sensors such as PPG, skin temperature, HR, and GSR. These sensors will offer a comprehensive understanding of AD progression, facilitating a robust early detection system. The development of wearable technology, incorporating these advancements, could empower individuals with spinal cord injuries, promoting independence and improving their quality of life.

### Supplementary Information

Supplementary Figures.

## Data Availability

The datasets used and/or analyzed during the current study are available from the corresponding author upon reasonable request.
