# Supplementary material for: Advancing spinal cord injury care through non-invasive autonomic dysreflexia detection with AI
Source: Sci Rep. 2024 Feb 10;14:3439. doi: 10.1038/s41598-024-53718-5 (PMC10858945; doi:10.1038/s41598-024-53718-5)
Supplement: Supplementary file 1 — Supplementary Figures. [file 41598_2024_53718_MOESM1_ESM.pdf]

Advancing Spinal Cord Injury Care through Non-Invasive Autonomic Dysreflexia Detection with AI

Supplementary material

To advance the detection and monitoring of autonomic dysreflexia (AD) in individuals with spinal cord injuries, we comprehensively analysed various features derived from skin nerve activity (SKNA). The primary objective was to evaluate the efficacy of these features in distinguishing between AD and non-AD events, shedding light on their potential as reliable markers for classification, as presented in Figure 1.

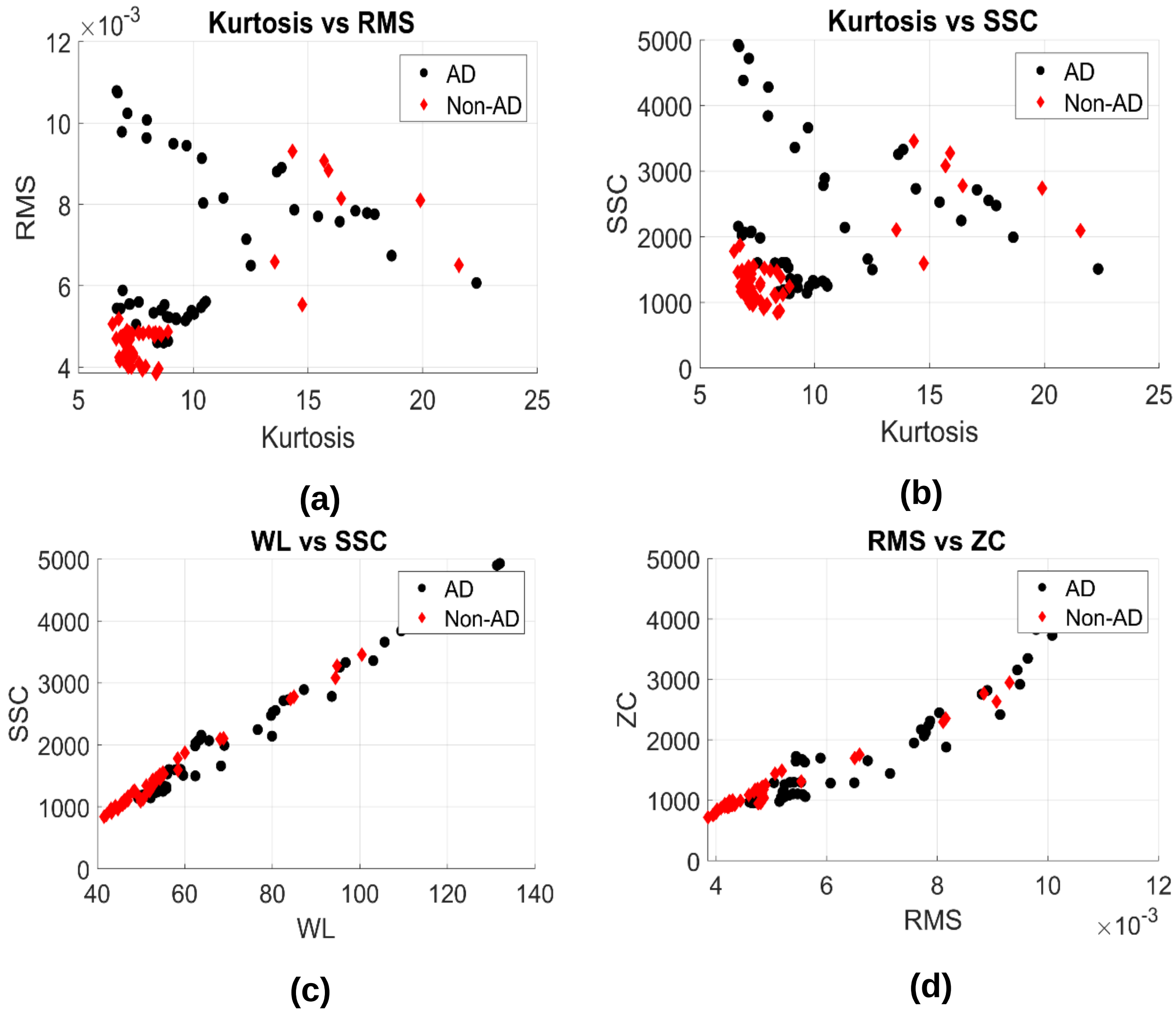

Supplementary Figure 1: Comprehensive Analysis of SKNA Features for Advancing AD Detection and Monitoring in Spinal Cord Injury Individuals. The scatter plots (a) Kurtosis vs RMS, (b) Kurtosis vs SSC, (c) WL vs SSC, and (d) RMS vs ZC showcase the intricate relationship between various SKNA features. Our primary objective was to assess the efficacy of these features in discerning between adverse events (AD) and non-AD events, unveiling their potential as robust and reliable markers for classification.

Further, in a comprehensive correlation analysis, we explored the intricate connections among key features linked to AD and non-AD counterparts. The outcomes are encapsulated in two revealing matrices illuminating the dataset's complex interrelationships as shown in Figure 2

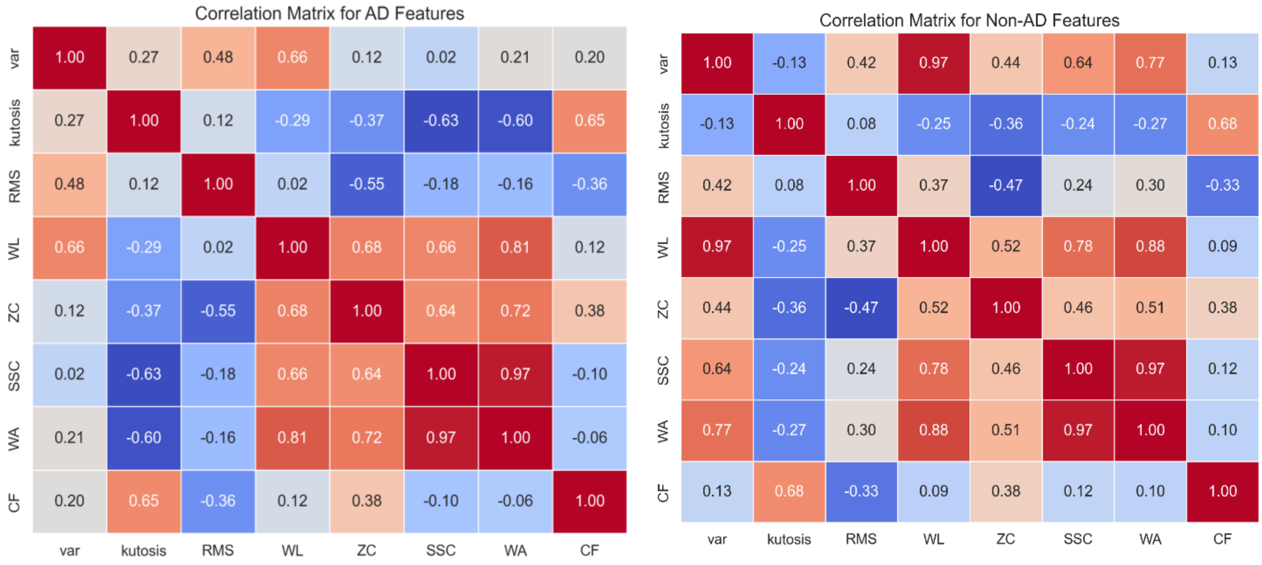

(a) **(b)**

Supplementary Figure 2: (a) Unravelling feature connections in AD events: A comprehensive correlation matrix. (b) Navigating the relationships in non-AD events: Insights from a revealing correlation matrix.
